# Incidence of treatment for postabortion complications in India, 2015

**DOI:** 10.1136/bmjgh-2020-002372

**Published:** 2020-07-19

**Authors:** Susheela Singh, Rubina Hussain, Chander Shekhar, Rajib Acharya, Melissa Stillman, Ann M Moore

**Affiliations:** 1 Research, Guttmacher Institute, New York, New York, USA; 2 Fertility Studies, International Institute for Population Sciences, Mumbai, India; 3 Population Council, New Delhi, India

**Keywords:** hospital-based study, maternal health, public health, treatment, descriptive study

## Abstract

Abortion has been legal under broad criteria in India since 1971. However, access to legal abortion services remains poor. In the past decade, medication abortion (MA) has become widely available in India and use of this method outside of health facilities accounts for over 70% of all abortions. Morbidity from unsafe abortion remains an important health issue. The informal providers who are the primary source of MA may have poor knowledge of the method and may offer inadequate or inaccurate advice on use of the method. Misuse of the method can result in women seeking treatment for true complications as well as during the normal processes of MA. An estimated 5% of all abortions are done using highly unsafe methods and performed by unskilled providers, also contributing to abortion morbidity. This paper provides new representative abortion-related morbidity measures at the national and subnational levels from a large-scale 2015 study of six Indian states—Assam, Bihar, Gujarat, Madhya Pradesh, Tamil Nadu and Uttar Pradesh. The outcomes include the number and treatment rates of women with complications resulting from induced abortion and the type of complications. The total number of women treated for abortion complications at the national level is 5.2 million, and the rate is 15.7 per 1000 women of reproductive age per year. In all six study states, a high proportion of all women receiving postabortion care were admitted with incomplete abortion from use of MA—ranging from 33% in Tamil Nadu to 65% in Assam. The paper fills an important gap by providing new evidence that can inform policy-makers and health planners at all levels and lead to improvements in the provision of postabortion care and legal abortion services—improvements that would greatly reduce abortion-related morbidity and its costs to Indian women, their families and the healthcare system.

**Key questions**

**What is already known?**

- About 16 million abortions take place in India annually, of which 75% occur outside of health facilities, mainly through medication abortion.
- A recent study on nine states in India estimated that 67% of abortions reported by women are unsafe; however, women greatly under-report abortions and this estimate is likely to be non-representative.
- Facility-based data provide alternative measures of the extent of unsafe abortion—the number and rate of women treated annually for abortion complications.

**What are the new findings?**

- In 2015, an estimated 5.2 million women received treatment for induced abortion complications nationally, a treatment rate of 15.7 per 1000 women aged 15–49, and this rate varies widely among the six states covered by the study.
- Approximately half of postabortion patients were treated for incomplete abortion resulting from use of medication abortion; many of these patients may not have needed treatment to complete their abortion.

**What do the new findings imply?**

- The high rate of women treated for abortion complications implies that it is critical that government policies address poor access to safe abortion care.
- The high proportion of postabortion patients treated for incomplete abortion from use of medication abortion indicates urgent need to help women get adequate information to use medication abortion safely.

## Introduction

Abortion has been legal in India since 1971 under broad criteria, including economic or social necessity, rape, incest, fetal impairment or contraceptive failure within marriage. Consent for the abortion is not required from the woman’s husband or from other family members, however, a guardian’s consent is required if the woman seeking an abortion is either younger than 18 or mentally ill. The act allows an unintended pregnancy to be terminated up to 20 weeks’ gestation; however, if the pregnancy is beyond 12 weeks, a second doctor’s approval is required. There are exceptions to this: If the provider is of the opinion that an abortion is immediately necessary to save a woman’s life, the gestational age limit does not apply and the second opinion is not required.^1 2^ However, access to safe and legal abortion services remains poor. Only obstetrician gynaecologists and doctors with a Bachelor of Medicine and Bachelor of Surgery who have been trained and registered to provide abortion are legally permitted to provide surgical abortion at approved abortion facilities. Registered providers in unapproved facilities may provide medication abortion (MA), but they must have referral linkages to approved facilities.^3 4^ Induced abortion provision is permitted at all government-run facilities at the Public Health Centre and higher levels, as long as the provider is registered in abortion provision. However, several factors contribute to the inadequacy of access to public sector abortion services, including high proportions of public facilities lacking trained staff and necessary equipment and supplies.^5–11^ Private facilities must be registered to provide this service and there exist substantial barriers to obtaining registration.^12^ The fact that private and, to some extent, public sector facilities and providers are concentrated in urban areas while a majority of the population is rural, also limits access. In addition, poor quality of care in facilities including the fact that some providers impose requirements based on their personal biases or beliefs may contribute to women’s preference for informal providers over facility-based services. Lack of trained providers, equipment and supplies, and barriers to registration among private providers, are some other barriers commonly reported by health facilities.^13^ These are just some of the reasons why access to legal abortion services remains limited, contributing to women opting for abortions from the informal sector.

Self-use of MA (Referred to as Medical Methods of Abortion (MMA) in India)—the combined regimen of mifepristone and misoprostol—that is acquired from chemists and informal vendors without a prescription, has become the principal method of abortion used in India.^14^ Since MA was approved as a method of abortion in India in 2003, it has become increasingly available in the country, in the form of ‘combipacks’ that contain dosages of the two medications for abortions up to 9 weeks gestation. In 2015, MA acquired outside of health facilities without a prescription accounted for over 70% of all abortions, while an estimated 5% of all women having abortions resorted to highly unsafe methods with a much higher probability of health complications compared with MA.^14–16^ When quality medications are used and recommended clinical protocols are followed correctly, 95%–98% of women using combipacks will have a complete abortion without complications within 9 weeks gestation.^17 18^ However, the main sources of MA in India are chemists and informal vendors who have poor knowledge of the method and provide little or no information, or inaccurate information, about use of the method.^19–21^ Such providers may not assess a woman’s gestational age (or assess it incorrectly), may fail to advise on how to take the medication, and may not provide accurate information on how the method works, how to recognise a complication or where to seek medical care should a complication occur. In addition, when male partners or other proxies purchase the medication, even if the seller has provided medically accurate information to the buyer, the instructions may not be accurately conveyed to the woman using the medication. These circumstances may result in morbidity due to method failure, incorrect use of MA or because of the use of poor quality medication.^22^ Furthermore, given inadequate access to information about what to expect, or due to gaps in provider knowledge or treatment protocols, some women are likely to receive unnecessary treatment when the abortion process is proceeding normally.^19 20^

A few studies have addressed the issue of morbidity from induced abortion in India. Some were conducted 15 or more years ago, and do not reflect current conditions, while others are more recent but focus on specific facilities or areas, and are not broadly generalisable.^23–27^ A recent study that used the large-scale Indian Annual Health Survey (data for 2010–2013) classified 67% of self-reported abortions in nine states as unsafe (Abortions were classified as unsafe if they were not performed or completed in a health facility, not performed or completed by a skilled birth attendant, or performed or completed at 20 weeks of gestation (~5 months) or beyond). It is important to bear in mind the limitations of that study: using self-reported experiences means that abortions are highly under-reported (due to stigma) and very likely to be non-representative. Additionally, it classified all MA abortions done outside of facilities as unsafe, meaning that the study is using a conservative measure of unsafe abortion,^28^ rather than WHO more nuanced classification of less safe and least safe.^29^

Little is known about the extent to which women in India currently obtain postabortion care (PAC) in facilities or the types and severity of postabortion complications for which women seek treatment. This paper addresses this evidence gap and presents findings from a large-scale study of health facilities conducted in 2015 in six states of India—Assam, Bihar, Gujarat, Madhya Pradesh, Tamil Nadu and Uttar Pradesh. Together, these states comprise about 45% of the population of women of reproductive age in India.^30^ This paper focuses on two key indicators related to abortion morbidity: the number and rate of women treated for complications resulting from induced abortion, and the type of complications women experience.

## Methods

The data for this analysis are from a primary data collection effort implemented by the authors and a study team: A Health Facility Survey (HFS) was conducted in a representative sample of 4001 public and private health facilities capable of providing postabortion services in six states of India: Assam, Bihar, Gujarat, Madhya Pradesh, Tamil Nadu and Uttar Pradesh, in 2015. Data are weighted to obtain regional and national estimates of the number of women receiving PAC in facilities

### Sample design

The study states were selected to represent the six major regions defined by the National Family Health Survey. We considered a number of demographic, economic and sociocultural indicators in selecting states, prioritising those indicators that are related to healthcare access and unintended pregnancy and abortion. These include the per cent of the population that is urban, women’s educational attainment, unwanted fertility, contraceptive prevalence and the per cent of women with unmet need for family planning.^31^ Our goal was to select states that were closer to the average for their region of these key indicators. We also prioritised selection of states with large populations, to maximise direct representation, and to minimise the impact of intraregional variation among states. The six selected states contain 45% of women of reproductive age in the country as a whole, providing a firm basis for developing national estimates. The selected states represent substantial proportions of reproductive aged women in their respective regions, varying from 29% for Tamil Nadu in the South region to 73% for Madhya Pradesh in the Central region.

Within each of these six study states, a representative sample of public and private facilities capable of providing PAC services was selected. The sample of public facilities (District Hospitals, Subdivisional Hospitals, Community Health Centres, Primary Health Centres, Medical Colleges and Employees’ State Insurance Corporation hospitals) was based on listings obtained from the government of India. No list existed for private facilities (hospitals, nursing/maternity homes and clinics), or for a few small categories of public sector facilities capable of providing PAC (‘Other Public’ types of facilities included the following: railway and tea hospitals, urban health centres as well as a few other facilities not categorised.) so the study team identified facilities of these types that provide abortion or postabortion services via a listing exercise. A sample was then drawn from this universe for each of these types of facilities. The sample design of the HFS also provides for representation of health facilities located in urban and rural areas, proportional to their relative contribution to the universe of facilities, within sector (public and private) and type of facility. Across six states, a total of 4001 public and private healthcare facilities were sampled (online supplementary appendix 1). Sample weights adjusted for the proportions sampled for each type of facility. The listing exercise, sample design and sample weights are described in greater detail elsewhere.^31^

10.1136/bmjgh-2020-002372.supp1 Supplementary data

### Data collection and analysis

For each sampled facility, the HFS was administered to one person who was knowledgeable about its provision of PAC. The interviews were conducted face to face by trained staff of data collection agencies that were contracted to implement the survey. Verbal answers were recorded by the interviewers on a paper survey.

The survey collected data on the number of postabortion patients treated, and the proportion of patients with each type of complication. Other approaches to obtaining these data (eg, extraction of data from facilities’ logbooks or other records of services provided) were considered; however, a high proportion of facilities do not maintain records, and the proportion that do so is highly variable by type of facility. The HFS asked respondents about the availability of logbook data and where available, interviewers extracted data on the number of induced abortion and PAC cases for the last 3 months. Only 4%–5% of facilities had logbook data in Bihar and Uttar Pradesh; 24%–29% in Gujarat and Madhya Pradesh; and 44% and 55%, respectively, in Tamil Nadu and Assam. Public facilities were the most likely to have logbooks and private facilities of any type were least likely. This paper presents results on the number and rate per 1000 women of reproductive age (15–49) treated for postabortion complications and the types of complications they experienced.

To obtain each facility’s PAC caseload, the HFS asked the respondent to estimate the number of women who were treated in the past month or year, and in the average month or year, for complications resulting from either induced abortion or miscarriage. It is difficult for providers to determine whether a complication is due to an induced abortion or a miscarriage especially for the large number of PAC cases that are admitted for incomplete abortion. Based on these data, we estimated annual caseloads for the past and average year, and averaged them to obtain the best estimate of the total annual number of PAC cases treated in each facility. The number of PAC cases treated in facilities at the state level, by type of facility and ownership were calculated, applying sample weights.

We used results from the six states to estimate the total number of women treated for postabortion complications in the country. This estimation involved the following assumptions: (1) that each surveyed state represents all non-surveyed states in their respective regions; (2) lacking data on the total count of private and ‘other public’ facilities for non-surveyed states, we used the ratio of women of reproductive age per each of these facility types in surveyed states to estimate the total number of these facilities in non-surveyed states in their respective regions and (3) that average caseloads for each type of facility in the surveyed states represent the average caseload for the same facility type in non-surveyed states. We created estimates of PAC caseloads for the six regions based on these assumptions and summed them to create a national estimate of the PAC caseload. More details on how results from six states were scaled up to the national level are provided elsewhere.^31^

To estimate the number of PAC cases resulting from induced abortion only, we applied an indirect estimation technique. We assume that early miscarriages will rarely receive treatment in a health facility. Based on clinical studies, the equivalent of 3.41% of the total number of births in a given year can be used to estimate the number of miscarriages at week 13 or higher gestations that would likely need treatment in a health facility.^32 33^ Taking into account the fact that not all women who have second-trimester miscarriages will get treatment, we assume that the proportion of these women who receive needed care in a facility is equivalent to the proportion of women who deliver in a health facility.^34^ We subtract the estimated number of women treated for complications from second trimester miscarriages from the total number of PAC cases, to obtain an estimate of the number treated for complications from induced abortion.

In the HFS, the respondent was asked to estimate the proportion of postabortion patients treated in their facility with each of seven types of abortion complications, explaining that the types were not exclusive because women may have more than one type of complication. The categorisation by type is derived from the WHO’s detailed International Classification of Diseases systems (ICD-9 and ICD-10).^35 36^ Simplified versions are used by researchers where study objectives are adequately served by a simpler approach that is feasible to implement. We applied a categorisation that was developed for purposes of estimating health systems costs of treatment of unsafe abortion.^37 38^ In the case of India, we added a category (incomplete abortion from MA/MMA) in implementing the simpler system. This was the first such attempt to measure the number of women who may be obtaining treatment in health facilities for incomplete abortion due to MA.

The first complication type respondents were asked about, ‘incomplete abortion from MA,’ is considered to be from induced abortion given the wording of the question; the second complication type, ‘incomplete abortion from any other procedure,’ suggests that this type of complication is also mainly from induced abortion, given the use of the word procedure in the question. The third complication type, ‘prolonged or abnormal bleeding,’ could result from induced abortion, but also may result from a miscarriage, and given that the time-period and amount of bleeding is not specified, the severity of this complication may vary. Infection of the uterus and surrounding area is likely a result of pregnancy termination, and could be very severe or moderately severe depending on the accompanying clinical symptoms.^39^ The remaining three types of complications experienced by PAC patients capture severe trauma that is highly likely to come about from interference with the pregnancy (rather than being a symptom of miscarriage): injury or perforation or laceration, sepsis and shock. We present the proportions with each type of complication as reported by HFS respondents. (Online supplementary appendix 2 contains the HFS questions on PAC).

10.1136/bmjgh-2020-002372.supp2 Supplementary data

### Patient and public involvement

The study did not directly involve patients, but is designed with the goal of benefiting the health of women of reproductive age. Public involvement was achieved through an advisory committee and meetings with individual stakeholders. The research questions and study design were informed by guidance from an advisory committee representing a range of civil society representatives—advocates, providers, researchers—as well as the public sector through representatives of the Ministry of Health and Family Welfare. In addition, the study team met individually with a number of individual stakeholders involved in women’s organisations, associations of obstetricians and gynaecologists, and groups that advocate for women access to sexual and reproductive healthcare, to obtain their guidance on the highest priority research questions that needed to be addressed. The advisory committee also provided input on analysis, findings and implications of the study.

## Results

### Incidence of facility-based PAC treatment at the state level

The number of women treated for induced abortion complications ranges from about 51 000 in Assam to 1.098 million in Uttar Pradesh, the most populous state of India (table 1). Results show relatively low treatment rates for three of the six study states (3.9 per 1000 women of reproductive age to 6.6 in Assam, Gujarat and Tamil Nadu), a substantially higher rate in Bihar (11.8), and much higher rates in Uttar Pradesh and Madhya Pradesh (21.3 and 26.2, respectively). Online supplementary appendix 1, table 2 present SEs and CIs for the total number of women treated for complications of either miscarriage or induced abortion: These estimates provide a rough indication of the likely range around measures of treatment for induced abortion complications. It is not feasible to calculate sample variance parameters for the latter indicators, because a number of external assumptions underlie calculation of the measures of treatment for complications from induced abortion.

10.1136/bmjgh-2020-002372.supp3 Supplementary data

**Table 1:** Number of women aged 15–49, number and rate of women treated for either miscarriage or induced abortion complications, and for induced abortion complications, six states of India, 2015

State	No of women 15–49	Treatment for miscarriage or for induced abortion complications		Treatment for induced abortion complications*	
		No treated	Treatment rate†	No treated	Treatment rate†
Assam	8 762 698	66 636	7.6	50 745	5.8
Bihar	25 321 313	360 457	14.2	299 766	11.8
Gujarat	17 048 928	105 905	6.2	67 108	3.9
Madhya Pradesh	19 385 375	559 507	28.9	508 532	26.2
Tamil Nadu	21 603 122	183 338	8.5	143 361	6.6
Uttar Pradesh	51 600 698	1 224 352	23.7	1 097 979	21.3

*An indirect estimation technique is used to separate patients treated for complications from miscarriage.

†The rate is the number treated per 1000 women aged 15–49.

### Types of postabortion complications

In all six study states, respondents estimated that a variable but high proportion of all women receiving PAC were admitted with incomplete abortion from use of MA—ranging from 33% in Tamil Nadu to 65% in Assam and is between 47% and 59% in the remaining four states (table 2). The second most common type of complication is prolonged or abnormal bleeding—27%–44% of PAC patients in five of the six states, although the proportion was much lower in Assam (15%). Prolonged bleeding and MA-related incomplete abortion are likely to be overlapping categories.

**Table 2:** Among postabortion patients (treated for complications of induced abortion or miscarriage) per cent estimated by facility respondents to be treated for specific types of complications, by state, 2015

Type of complication*	Assam	Bihar	Gujarat	Madhya Pradesh	Tamil Nadu	Uttar Pradesh
Incomplete abortion from MA	65	51	48	47	33	59
Incomplete abortion from any other procedure	17	32	22	21	23	25
Prolonged/abnormal bleeding	15	30	31	44	27	32
Infection of uterus/surrounding areas	4	16	9	10	12	15
Injury/perforation/laceration	2	9	3	3	6	4
Sepsis	3	5	4	3	7	5
Shock	1	4	3	2	3	3

*A patient may have more than one type of complication; the per cent values do not sum to 100% for each state.

MA, medication abortion.

Incomplete abortion resulting from use of methods other than MA was also common, ranging from a low of 17% in Assam to a high of 32% in Bihar. Notable minorities of women treated for abortion complications had very serious symptoms, indicative of unsafe induced abortions done with invasive methods. For example, the proportion with infection of the uterus and surrounding areas ranged from 9% to 16% in five of the six states; Assam was again an outlier (4%). The percent with sepsis, a more serious complication, was also substantial, at 3%–7% across the six states. Because of potential overlap, it is not possible to estimate precisely how many women are treated for severe complications overall. However, the proportion of women who experienced infection of the uterus and surrounding areas—the largest group among the four types of severe complications—can be considered a minimum estimate of the proportion with such complications.

If we assume that all PAC patients who are admitted with incomplete abortion from use of MA would have successfully completed the abortion under better service provision conditions and not needed medical care, the rate of treatment for postabortion complications would be greatly reduced. The impact on the treatment rate would not be exactly proportional to these percentage values, due to some converse influences. First, there is some overlap between categories of abortion complications, and some women classified as having incomplete abortion from use of MA may also have had another type of complication. Second, the clinical efficacy of MA is 95%–98% when the method is used correctly and drugs are of high quality, and at a minimum 2%–5% of all users of MA would have incomplete abortions and may need medical treatment. Since the large majority of MA users in India are obtaining the method from informal vendors, their likelihood of experiencing an incomplete abortion or other complications would be much higher than the clinical failure rate, for example, because the medication was self-administered incorrectly, because the medication was compromised or because they were not given the correct dosage for their gestation. Nevertheless, the impact of ensuring that all women who use MA (and especially those who obtain it outside of facilities) receive accurate information and good quality MA supplies would substantially reduce women’s experience of complications and need for facility-based treatment.

### Understanding state variation in incidence of treatment for postabortion complications

The range in the incidence of treatment for PAC may be related to the abortion rate (other factors being equal). However, the per cent of all abortions that are treated for complications in facilities do not show a consistent relationship with the abortion rate; it ranges widely from 8%–9% in two states to 20%–24% in two states and even higher proportions in two states (35%–46%; table 3). Similarly, it may be expected that the proportion of all abortions that occur in facilities (which are presumably safer than those occurring outside the formal health system) is related to the proportion of all abortions that are treated for complications. However, the data do not show a systematic relationship between the proportion of abortions occurring in facilities and the likelihood of complications among all abortions; this is likely because facility-based abortions are a minority of abortions in all six states (11%–32%, table 3).

**Table 3:** Number of induced abortions and abortion rate; per cent of induced abortions that have complications treated in a health facility; treatment rate; per cent of MA abortions treated for complications in facilities; and per cent distribution of all abortions by source, by state, 2015

Indicator	Assam	Bihar	Gujarat	Madhya Pradesh	Tamil Nadu	Uttar Pradesh
No and rate per 1000 women aged 15-49						
Total no of induced abortions	580 054	1 250 958	811 835	1 109 951	707 938	3 151 589
Induced abortion rate	66.2	49.4	47.6	57.3	32.8	61.1
Abortion treatment						
Per cent of all induced abortions that were treated for complications in facilities	8.8	23.9	8.2	45.8	20.1	34.9
Treatment rate per 1000 women 15-49 for complications of induced abortion	5.8	11.8	3.9	26.2	6.6	21.3
Medication abortion treatment						
Of all women who had a medication abortion, % who are treated in facilities for incomplete abortion due to use of MA	9.7	17.7	7.4	29.6	11.2	26.2
Per cent distribution of all abortions by source						
% of abortions occurring in health facilities	21.1	15.5	15	25.5	32.3	11.4
% of abortions occurring as MA outside of facilities	73.8	79.3	79.8	69.4	62.6	83.4
% of abortions that are ‘other’ (non-facility/non-MA)	5.2	5.2	5.2	5.2	5.2	5.2

MA, medication abortion.

Notably, treatment for incomplete abortion due to use of MA comprises a relatively small proportion of all women who used MA (combining those who obtained the method within facilities with those who obtained it outside of a health facility) in three states (7%–11% in Assam, Gujarat and Tamil Nadu, table 3). This proportion is somewhat higher in Bihar (18%), and much higher in Uttar Pradesh and Madhya Pradesh (26% and 30%, respectively). This wide range across states is likely influenced by two opposing factors: access to treatment and how well MA is used.

### National estimate of treatment for induced abortion complications

The total number of women treated for complications resulting from induced abortion based on scaling up results from these six states to their respective regions is 5.2 million, and the rate is 15.7 per 1000 women of reproductive age per year. As discussed above, approximately half of the women treated for abortion complications are estimated to be treated for complications of incomplete abortion resulting from use of MA, many of whom may not have needed treatment to complete their abortion. If all the women in this category were assumed to have not needed treatment, the national number of women with abortion complications needing care would be 2.4 million, with a corresponding rate of 7.2 per 1000 women of reproductive age (In both scenarios, the estimated number and rate are underestimates because an unknown number of women have complications and do not get needed care). While the latter rate is purely hypothetical as some will need treatment even under perfect use conditions, it is useful for highlighting the potential impact of correct knowledge and use of MA on the number and rate of facility-based complications of induced abortion.^40^

With respect to the severity of complications, taking the same approach discussed above for state-level estimates, we estimate that the minimum national number of women with severe complications is 330 000 annually, based on the proportion of PAC patients treated the most prevalent of the severe complications (infections of the uterus and surrounding areas). Since some women who are treated for the other three severe complications would not overlap with the group treated for infections of the uterus and surrounding areas, the total number of women with severe complications is likely higher than 330 000.

## Discussion

This study presents new, representative data on the magnitude of treatment for postabortion complications and the types of complications in India—data that can inform policies and programmes to provide comprehensive postabortion services.

Despite broad legal criteria under which abortion is permitted in India, its postabortion treatment rate is high at 15.7 per 1000 women 15–49. By comparison, the developing world average treatment rate was 6.9 per 1000 women 15–44 in 2012.^40^ The rate for India is closest to treatment rates for Pakistan (treatment rate of 13.9), where the abortion rate is similar (50 per 1000 women), but where abortion law is highly restrictive, and unsafe abortion is likely to be prevalent.^41^ Bangladesh and Nepal, countries with a mixture of unsafe and safe procedures (menstrual regulation and legal abortion, respectively), have lower treatment rates, 6.1 and 8.2, respectively.^42 43^ India’s treatment rate is disproportionately high relative to its abortion rate by comparison with the latter two countries, with abortion rates of 39 and 42, respectively.

The PAC treatment rate varies widely among the six study states, from a very low rate of 3.9 in Gujarat to rates similar to the world average in Assam and Tamil Nadu (5.8 and 6.6, respectively), and higher rates in the other three states: a rate of 11.8 in Bihar and rates that are at least three times as high as the world average in two states (21.3 in Uttar Pradesh and 26.2 in Madhya Pradesh). While removing all women treated for incomplete abortion from MA would result in a treatment rate of 7.2 nationally, the true rate of induced abortion complications requiring treatment is likely to be higher than this hypothetical rate but lower than the overall national estimate of 15.7 cases per 1000 women. One factor that may contribute to the differential treatment rate between India and other countries is the much higher level of use of MA obtained informally in India. More research is needed to understand women’s medical status on admission and the pathways through which they sought PAC. Representative evidence is lacking on the proportion of women admitted for PAC who did not need treatment to complete the abortion because it was a medical abortion in progress that would have completed following the normal process.

The study has several limitations. The estimates are based on respondents’ reports of PAC treatment for their facilities, and are not patient specific. We rely on the perception of the respondent to estimate caseloads and proportions with different complications. The data presented do not capture women who needed and did not obtain care for complications. Our estimates for miscarriage are based on assumptions that rely in part on clinical studies that establish the biological pattern of pregnancy loss by gestation, and these studies are not specific to India. In addition, the national estimate of the number and rate of women treated for abortion-related complications relies on several assumptions: that the average caseload of PAC treatment in surveyed facilities represents the average caseload in facilities of the same type/size and ownership in states that were not surveyed; and in addition, for the private sector, that the ratio of population per facility in surveyed states represents the situation in non-surveyed states. Future research that measures uncertainty around these assumptions and studies on care seeking for miscarriage are needed.

While it is useful to explore variation in the treatment rate across the six states, including indicators that may relate to this outcome at the state level, intrastate variation may be as important as interstate differences. Future research on variation between smaller geographical areas, and in differences between women who live in urban and rural areas, on both the outcome measure (the rate of treatment for abortion complications) and on explanatory variables—would strengthen our understanding of determinants of women’s access to PAC. In addition, some MA products sold are possibly substandard and would not have been effective even if used correctly^44^; research is needed to ascertain whether, and the extent to which, this is happening.

Abortion is a very safe procedure whether surgical or medication, and detailed guidelines have been issued by WHO.^17 45^ The relatively high incidence of women being treated for postabortion complications in India has important implications for policies and programmes. Some key implications are: increasing access to safe and legal abortion in health facilities; implementing strategies to enable women to safely access and use MA acquired outside of health facilities.

Access to abortion services in health facilities can be improved by:

- Adequately equipping and staffing facilities that are permitted to provide abortion.
- Improving the quality of abortion care in health facilities--addressing a range of issues such as inadequate privacy and confidentiality, provider biases and cost barriers.
- Standardising and streamlining the process for registering private facilities, and ensuring that efficient processes are in place and implemented.
- Amending the Medical Termination of Pregnancy Act (2003) to permit midlevel providers such as nurses, midwives and other similar cadres of professionals to provide abortion services, accompanied with a plan for training these providers to do so.
- Equipping health facilities to meet WHO recommendations (also included in the Medical Termination of Pregnancy Act) on privacy and confidentiality.

- Addressing provider bias towards women who have abortion complications through training and support.
- Reducing cost barriers by enforcing the policy that public health facilities not charge women for services.

There are several strategies to enable women to access MA and safely self-manage its use. These include:

- Changes in guidelines to permit and train additional cadres of healthcare professionals to provide MA.
- Training vendors of MA in essential aspects of MA use so that they can appropriately advise women, and provide accurate information to users.
- Adopting (and adapting as needed) approaches that have been successfully applied in other countries to enhance effective self-use of MA such as hotlines offering medically accurate advice.
- Improving the inserts in the MA packets to help facilitate women’s ability to use the method correctly (eg, making sure the information is written in a language that the woman understands; including graphics, etc), what to expect and when to seek care.
- Developing innovative approaches for public dissemination of information about correct use of the method by means that reach the majority of women (and these will likely vary depending on the context within and across states).

Such advances would provide women with a range of safe abortion choices, improve the safety of abortion, and reduce postabortion complications and their health consequences and costs to women, families and the health system.

## Conclusion

This article contributes to filling a critical evidence gap. It shows that the rate of treatment for abortion complications in India is higher than that typically found in countries with highly restrictive abortion laws, despite the broad legal grounds under which abortion is permitted. In addition, the evidence on types of complications women present with at facilities helps elucidate the severity of morbidity, as well as the types of interventions and medical resources that are needed to provide appropriate treatment. The large majority of women having abortions currently use MA obtained outside of facilities: They need accurate information to help them use MA correctly and to understand the normal process of an MA.^19 20 46^ This study provides new information for policy-makers and programme planners that highlight the need for improving women’s access to safe and legal abortion services in order to reduce abortion-related morbidity and mortality, improvements that would greatly benefit women’s health and survival.
