# Supplementary material for: Incidence of treatment for postabortion complications in India, 2015
Source: BMJ Glob Health. 2020 Jul 19;5(7):e002372. doi: 10.1136/bmjgh-2020-002372 (PMC7371025; doi:10.1136/bmjgh-2020-002372)
Supplement: Supplementary data [file bmjgh-2020-002372supp001.pdf]

Appendix 1: Table 1. Number of facilities included in Health Facilities Survey, by type and state, 2015

Facilities	Assam	Bihar	Gujarat	Madhya Pradesh	Tamil Nadu	Uttar Pradesh	All
**Public**	150	320	262	383	393	538	2,046
Hospitals	35	65	52	96	99	74	421
Community health centres	28	37	71	81	86	144	447
Primary health centres	78	210	137	186	178	263	1,052
"Other Public"*	9	8	2	20	30	57	126
**Private**	46	337	218	277	393	684	1,955
Hospitals	19	83	163	136	222	214	837
Nursing and maternity homes	19	104	39	83	84	101	430
Clinics	8	150	16	58	87	369	688
**Total**	**196**	**657**	**480**	**660**	**786**	**1,222**	**4,001**

* This group includes the following types of public sector facilities: railway and tea hospitals, urban health centers as well as a few other facilities.
