# Supplementary material for: Incidence of treatment for postabortion complications in India, 2015
Source: BMJ Glob Health. 2020 Jul 19;5(7):e002372. doi: 10.1136/bmjgh-2020-002372 (PMC7371025; doi:10.1136/bmjgh-2020-002372)
Supplement: Supplementary data [file bmjgh-2020-002372supp002.pdf]

Appendix 2: Health Facilities Survey: Questions on postabortion care

Section 4: Post Abortion Complications Care (PAC) Services

[Interviewer read:] *Now, I would like to ask you some questions about this facility's provision of services to treat postabortion complications, generally referred to as PAC services. Please include complications cases from both spontaneous abortions and induced abortions that may have been received here or* ***anywhere else****.*

Postabortion complications, as defined here, include a range of complications from extremely serious cases such as sepsis or a perforated uterus, to "incomplete abortions," which are usually identified by heavy bleeding, but nevertheless need medical attention.

[Interviewer: Please note that the abortion complications questions relate to both spontaneous and induced abortions. You should reiterate this as often as possible while completing this section.]

[Interviewer read]: *Now, I am going to ask you some questions about the actual numbers of women treated* ***at this facility*** *for complications resulting from both induced and spontaneous abortions. Kindly base your answers on your best estimate of the TOTAL number of women treated for PAC, whether or not all cases are entered into a register.*

**409**	In the **average month**, how many PAC patients would you estimate are treated as **outpatients** in this facility as a whole?		a. Number of PAC **outpatients** in the **average month**
	OR		OR
	**[Interviewer: Only if respondent can not provide an answer for the average month, ask:]** In the **average year**, how many PAC patients would you estimate are treated as **outpatients** in this facility as a whole? **[Interviewer: Specify that this is a full 12 months]**		b. Number of PAC **outpatients** in the **average year**
**410**	During the **past month**, how many PAC patients would you estimate were treated as **outpatients** in this facility as a whole?		a. Number of PAC **outpatients** in the **past month**
	OR		OR
	**[Interviewer: Only if respondent can not provide an answer for the past month, ask:]** During the **past year**, how many PAC patients would you estimate are treated as **outpatients** in this facility as a whole? **[Interviewer: Specify that this is a full 12 months]**		b. Number of PAC **outpatients** in the **past year**

**[Interviewer: See Q408. If the health facility provides ONLY outpatient services, go to Q 413.]**			
411	In the **average month,** how many PAC patients would you estimate are treated as **inpatients** in this facility as a whole?		a. Number of PAC **inpatients** in the **average month**
	OR		OR
	**[Interviewer: Only if respondent can not provide an answer for the past month, ask:]**		
	In the **average year**, how many PAC patients would you estimate are treated as **inpatients** in this facility as a whole?		b. Number of PAC **inpatients** in the **average year**
	**[Interviewer: Specify that this is a full 12 months]**		
412	During the **past month**, how many PAC patients would you estimate were treated as **inpatients** in this facility as a whole?		a. Number of PAC **inpatients** in the **past month**
	OR		OR
	**[Interviewer: Only if respondent can not provide an answer for the past month, ask:]**		
	During the **past year**, how many PAC patients would you estimate are treated as **inpatients** in this facility as a whole?		b. Number of PAC **inpatients** in the **past year**
	**[Interviewer: Specify that this is a full 12 months]**		
413			***Summary in an AVERAGE MONTH***
	Just to confirm, from what you have just told me, in an **average month (or average year)** this facility treats _____ outpatients and _____ inpatients for abortion complications, for a total of _____ patients. **Is this correct?**		a. # of outpatients
			b. # of inpatients
			c. Total
			OR
			Summary in an AVERAGE YEAR
	**[Interviewer: Please read out the total number of spontaneous and induced abortion complications (PAC) patients seen at this facility as outpatients (Q409) and as inpatients (Q411) in an average month (or year).]**		d. # of outpatients
			e. # of inpatients
			f. Total

	**[Interviewer: If not correct, then correct Q 409 and Q 411 and insert above.]**		
**414**	Just to confirm, from what you have just told me, in the **past month (or past year)** this facility treats _____ outpatients and _____ inpatients for abortion complications, for a total of _____ patients. **Is this correct?**		***Summary in PAST MONTH***
			a. # of outpatients
			b. # of inpatients
			c. Total
			OR<br>***Summary in PAST YEAR***
	**[Interviewer: Please read out the total number of spontaneous and induced abortion complications (PAC) patients seen at this facility as outpatients (Q410) and as inpatients (Q412) in the past month (or year).]**		d. # of outpatients
			e. # of inpatients
			f. Total
	**[Interviewer: If not correct, then correct Q 410 and Q 412 and insert above.]**		

[Interviewer read]: *Now, I would like to ask about the specific abortion complications that were treated at this facility in recent years.*

**417**	Thinking about 100 PAC cases **in general**, how many has this facility treated with the specific complication I will mention, keeping in mind one woman may have multiple complications?		# of PAC patients
			a. Incomplete abortion from MA/MMA
			b. Incomplete abortion from any other procedure
			c. Prolonged or abnormal bleeding
			d. Infection of the uterus/surrounding areas
	**[Interviewer: READ list out. Ask how many women receiving PAC out of every 100 had each complication mentioned. Total may add to more than 100 because women may have multiple complications. If facility has not treated anyone with that complication, fill in 0.]**		e. Injury/perforation/laceration
			f. Sepsis
			g. Shock (hemorrhagic/septic)
			x. Others, (specify): ____________________
