# Supplementary material for: Incidence of treatment for postabortion complications in India, 2015
Source: BMJ Glob Health. 2020 Jul 19;5(7):e002372. doi: 10.1136/bmjgh-2020-002372 (PMC7371025; doi:10.1136/bmjgh-2020-002372)
Supplement: Supplementary data [file bmjgh-2020-002372supp003.pdf]

Appendix 1: Table 2. Total number of postabortion complication cases treated, standard errors and confidence intervals around estimates, by state, 2015

State	Total annual postabortion complication cases treated	Standard error	95% Confidence Interval	
			low	high
Assam	66,636	8,031	52,558	80,714
Bihar	360,457	29,587	309,837	411,078
Gujarat	105,905	10,215	88,398	123,412
Madhya Pradesh	559,507	38,912	493,812	625,203
Tamil Nadu	183,338	20,893	148,394	218,281
Uttar Pradesh	1,224,352	132,107	1,003,333	1,445,372

Note: Postabortion care estimates are weighted and standard errors and 95% confidence intervals are calculated taking into account the complex sample design of the HFS. Finite population correction (fpc) adjustments were made to adjust for the correlation between the samples.
